# Optimizing bioremediation techniques for soil decontamination in a linguistic intuitionistic fuzzy framework

**DOI:** 10.1038/s41598-024-66863-8

**Published:** 2024-07-10

**Authors:** Hanan Alolaiyan, Misbah Hayat, Umer Shuaib, Abdul Razaq, Mohammed Abdullah Salman, Qin Xin

**Affiliations:** 1 Department of Mathematics, King Saud University, Riyadh, Saudi Arabia; 2 Department of Mathematics, Government College University, Faisalabad, 38000 Pakistan; 3 Department of Mathematics, Division of Science and Technology, University of Education, Lahore, 54770 Pakistan; 4 Department of Mathematics and Statistics, Amran University, Amran, Yemen; 5 Faculty of Science and Technology, University of the Faroe Islands Vestara Bryggja, 15, FO 100 Torshavn, Faroe Islands Denmark

**Keywords:** Linguistic intuitionistic fuzzy sets, Linguistic intuitionistic fuzzy Dombi aggregation operators, Multi-attribute decision making problem, Bioremediation techniques, Engineering, Mathematics and computing

## Abstract

Bioremediation techniques, which harness the metabolic activities of microorganisms, offer sustainable and environmentally friendly approaches to contaminated soil remediation. These methods involve the introduction of specialized microbial consortiums to facilitate the degradation of pollutants, contribute to soil restoration, and mitigate environmental hazards. When selecting the most effective bioremediation technique for soil decontamination, precise and dependable decision-making methods are critical. This research endeavors to tackle the aforementioned concern by utilizing the tool of aggregation operators in the framework of the Linguistic Intuitionistic Fuzzy (LIF) environment. Linguistic Intuitionistic Fuzzy Sets (LIFSs) provide a robust framework for representing and managing uncertainties associated with linguistic expressions and intuitionistic assessments. Aggregation operators enrich the decision-making process by efficiently handling the intrinsic uncertainties, preferences, and priorities of MADM problems; as a consequence, the decisions produced are more reliable and precise. In this research, we utilize this concept to devise innovative aggregation operators, namely the linguistic intuitionistic fuzzy Dombi weighted averaging operator (LIFDWA) and the linguistic intuitionistic fuzzy Dombi weighted geometric operator (LIFDWG). We also demonstrate the critical structural properties of these operators. Additionally, we formulate novel score and accuracy functions for multiple attribute decision-making (MADM) problems within LIF knowledge. Furthermore, we develop an algorithm to confront the complexities associated with ambiguous data in solving decision-making problems in the LIF Dombi aggregation environment. To underscore the efficacy and superiority of our proposed methodologies, we adeptly apply these techniques to address the MADM problem concerning the optimal selection of a bioremediation technique for soil decontamination. Moreover, we present a comparative evaluation to delineate the authenticity and practical applicability of the recently introduced approaches relative to previously formulated techniques.

## Introduction

### Background

In order to make a decision in a complicated and intricate environment, MADM entails methodically evaluating and weighing a number of essential variables. The use of aggregation operators (AOs) in MADM is recognized for their effectiveness in addressing practical difficulties in several fields, including natural and social sciences, environmental studies, engineering, and related areas. The prevailing assumption among individuals is that decision-making pertaining to an option is predicated upon the availability of precise numerical data elucidating the attributes of said option and the corresponding relevance of these traits. Aggregation operators enable the final aggregate output to include all individual values by combining multiple values into a single value inside a certain collection. During the past decades, the complexity of decision-making problems arising in the presence of social interactions has been rising, along with the need for the design of advanced theoretical frameworks and tools to enable the decision-maker to express preferences suitable to the case of ambiguity. In 1965, Zadeh^1^ introduced the concept of fuzzy sets as a way of dealing with imprecise information. In certain scenarios, exclusive reliance on membership degrees may inadequately capture the nuanced information inherent in practical problem domains. In an effort to address this issue, Atanassov^2^ provided a more sophisticated theoretical framework by defining IFSs, which enhanced the paradigm of fuzzy sets through the delineation of quantified degrees for both membership and non-membership aspects. From a technical standpoint, the summation of these degrees is subject to the limitation of being less than or equal to one in the framework of IFS. Despite the advancement and generalization of fuzzy set theory, its practical utility is constrained by the requisite precision in assigning membership or non-membership degrees to elements within the confined range of zero to one. Consequently, their utility is constrained to quantitative contexts, precluding adept handling of qualitative data. When communicating information in a quantitative framework, numerical representation is typically used. Precise numerical values are not a practical means of quantifying information in a qualitative setting, which is defined by the existence of subjective or ambiguous knowledge. In such scenarios, a pragmatic approach involves the substitution of numerical quantification with linguistic assessment. This entails the evaluation of variables in the problem domain through the application of linguistic terms. Diverse methodologies have been formulated to tackle problems characterized by the integration of linguistic information, particularly within the framework of MADM.

In 1975, Kahne^3^ developed a method of decision-making for circumstances where each option needs to be assessed according to several factors with varying levels of importance. Jain^4^ originally introduced a fuzzy optimal alternative computing method for decision-making in 1977. Dubois and Prade^5^ devised many operations on fuzzy sets in 1978. Certain AOs on fuzzy sets were presented by Yager^6^. Chen and Tan^7^ put forward the scoring functions for IFS in 1994. A technique for identifying approaches for facilitating group decision-making within the IFS framework was introduced in^8^. In 2005, Li^9^ proposed a number of models for linear programming and techniques for MADM in an IFS scenario. In 2006, geometric aggregation operations were defined within the IFS framework by Xu and Yager^10^. Later, in 2007, Xu^11^ created arithmetic AOs on IFS. To address the MADM issue, researchers developed some novel AOs for IFS^12^. The induced generalized AOs were suggested in^13^ for IFS and were subsequently applied in group decision-making settings. In 2014, Huang^14^ explored Hamacher AOs for IFS in order to develop a decision-making methodology. In addition, numerous practical solutions were developed to deal with the issue of various MADM problems after Verma^15^ introduced the Bonferroni mean operators on IFS in 2015. Moreover, a spectrum of effective strategies has been innovated to confront the challenges associated with diverse MADM problems, as elucidated in references^16–20^.

### Related work

In 1982, Dombi^21^ expounded upon a diverse array of fuzzy operators. Researchers found that using Dombi AOs is one of the best ways to tackle MADM difficulties. The researchers introduced spherical fuzzy Dombi AOs in reference^22^, whereas Pythagorean fuzzy Dombi AOs were discussed in reference^23^. The bipolar fuzzy Dombi AOs^24^, picture fuzzy Dombi AOs^25^, and q-rung orthopair fuzzy Dombi AOs^26^ were established. In^27^, Liu et al*.* introduced interval-valued fuzzy Dombi AOs for assessing the risk of information security. Additionally, Dombi operators were introduced on IFS by Seikh and Mandal^28^. In 2022, the hesitant T-spherical fuzzy Dombi AOs were presented by Karaaslan and Husseinawi^29^, along with their application in MADM. Moreover, many developments in the framework of the Dombi aggregation environment can be seen in^30–35^. In addition to studying the properties and semantics of linguistic term sets, Herrera and Herrera-Viedma^36^ developed a linguistic decision-making technique based on aggregation operations. Later, Xu^37^ proposed uncertain linguistic variables, which describe fuzzy data using a linguistic interval instead of one number. The authors^38^ developed Bonferroni mean operators for uncertain linguistic variables. Moreover, hesitant fuzzy linguistic term sets^39^, hesitant fuzzy linguistic AOs^40^, and hesitant fuzzy hybrid AOs^41^, were developed within the context of HFSs. The adaptability of IFSs within qualitative environments was further enhanced by Chen et al*.*^42^. This refinement introduces a fusion of IFSs and linguistic models, which account for both linguistic membership and non-membership degrees. The result is a capability for LIFSs to compete in apprenticeships in the uncertain and ambiguous data domain. A dedicated suite of AOs designed specifically for LIFSs, given the following: LIF weighted averaging (LIFWA), LIF ordered weighted averaging, LIF hybrid averaging, and their geometric counterparts in^43^, constitutes a principled framework to enhance the accuracy and capability of IFSs in a complex and qualitative domain. Ou et al.^44^ use the TOPSIS method for linguistic multi-criteria decision-making using LIF knowledge. Qiyas et al.^45^ defined linguistic intuitionistic cubic fuzzy AOs. Bai et al.^46^ articulated their work on incomplete intuitionistic fuzzy (IF) behavioral group decision-making based on multigranulation in 2023. In their 2023 work, Zhang et al.^47^ presented a very efficient approach for handling low-resource languages in intelligent decision systems. In 2024, Ding et al.^48^ proposed three-way decisions in a generalized IF environment. In 2024, Hussain et al.^49^ devised innovative IF Aczel-Alsina Hamy mean operators. Moreover^50–53^ encompasses noteworthy advancements in the IF environment.

Environmental pollution is a severe, pervasive global problem that negatively affects ecosystems as well as human health^54,55^. To tackle the problem successfully, highly efficient cleanup technologies need to be developed. The resolution of these issues requires the creation of exceptionally efficient remediation methods. Traditional methods, on the other hand, are not only limited but are also intrinsically incapable of providing precision. Therefore, in order to address the inherently multiscale and highly dimensional control problems at the level of environmental systems, an innovative strategy is required. Utilizing the inherent capabilities of biological agents, bioremediation emerges as an especially advantageous strategy for mitigating the adverse effects of environmental contaminants^56,57^. When the potential of bioremediation is disregarded, achieving optimum efficacy remains a formidable undertaking. Conventional optimization approaches might be inadequate for capturing the intricate dynamics that seem to form the fundamental basis of biological systems, given their complex nature.

### Motivations and objectives of the current study

The conceptual framework of IFS has had a significant impact on a variety of scientific and technological disciplines, such as engineering, medical diagnosis, information technology, pattern recognition, and decision science. The incorporation and impact of this extraordinary theoretical construct are self-evident due to its extensive and exhaustive integration into systems and practices. LIFSs are distinct from IFSs in that they address the degrees of both linguistic membership and linguistic non-membership simultaneously, whereas IFSs exclusively address the degrees of membership and non-membership. This fundamental characteristic of LIF settings enables a more nuanced approach to situations in which both membership and non-membership information are pertinent, as well as a more comprehensive understanding of uncertainty. The linguistic essence is integrated into the IF framework through the use of linguistic identifiers in specific sets. This offers a representation of information that is more easily comprehensible and interpretable to humans. This is highly beneficial, primarily due to the significant influence of qualitative assessments and linguistic expression on the decision-making process. LIFSs are more successful than IF environments as a result of this specific aspect.

Several researchers attempted to introduce a series of aggregation operators and information measures within the IF environment in order to address the complexities of MADM. However, these operators only manage membership and non-membership functions, which can occasionally be too complex and unclear for human comprehension. This motivates the development of improved aggregation operators for LIF settings, which can work better in cases when more qualitative interpretation is needed. A class of adaptable multi-functional aggregation operators known as the Dombi operators is particularly well-suited for capturing the inherent conflict that is a hallmark of operationality and decision-making. The synthesis of data is accomplished within this specific framework by means of systematic averaging procedures, which ultimately result in the development of a singular combined measure. It is crucial to recognize that the Dombi cultural ethos is characterized by its adaptability and ability to make prompt decisions. These operators demonstrate the highest degree of adaptability to MADM issues in the face of significant operational changes.

The following is a brief overview of the main contributions made to this study:

i. Two innovative aggregation operators, LIFDWA and LIFDWG, have been designed to address intricate decision-making scenarios that involve LIF data.
ii. The structural features of the suggested operators have been proven. This underscores the mathematical nature of these operators.
iii. A systematic approach to resolving MADM problems within the context of LIF knowledge is offered through the use of newly developed operators.
iv. The proposed methodology is applied to address a particular MADM problem related to the best choice of a bioremediation strategy for soil decontamination. This highlights the significance of LIFDWA and LIFDWG in the process of making decisions.
v. The effectiveness of the proposed methodology is assessed by a comprehensive comparison examination. The comparative findings indicate that the developed approach is reliable and consistent.

The following is the arrangement of this article's succeeding sections. In Section “Foundational Concepts Overview”, fundamental definitions that are essential for understanding the innovation inherent in the contributions of this article are outlined. In the context of the LIF, novel score and accuracy functions for solving MADM problems are described in Section “Formulation of Novel Score and Accuracy Functions for LIFNs”. The structural characteristics of LIF Dombi aggregation operators are detailed in Section “Dombi Aggregation Operators for LIFNs”. By employing the suggested approaches, Section “Utilization of Designed Aggregation Operators in MADM Framework” elucidates the sequential mathematical process underlying MADM challenges in the LIF setting and provides an example of the resolution to the dilemma of determining the most effective bioremediation method to purify soil that has been contaminated. A comparative analysis is incorporated to demonstrate the practicality and dependability of the newly developed methodologies in relation to well-established ones. The paper is concluded in Section “Conclusions”, which provides conclusive findings derived from the research that was presented.

## Foundational concepts overview

This section offers a summary of key definitions necessary for a full understanding of the subject under investigation in this study.

### Definition 1 (

^1^). A fuzzy set $B$ of universal set $X$ can be represented by $B=\left\{\left(x,{\mu }_{B}\left(x\right)\right)|x\in X \right\},$ ${\mu }_{B}: X\to \left[\text{0,1}\right]$ represents the membership function that characterizes the degree of membership for elements of $X$ to $B,$ such that $0\le {\mu }_{B}\left(x\right)\le 1.$

### Definition 2

(^2^). An IFS $C$ of $X$ is characterized as $C=\left\{\left(x,{\mu }_{C}\left(x\right),{\nu }_{C}\left(x\right)\right)|x\in X\right\},$ where ${\mu }_{C},{\nu }_{C}:X\to [\text{0,1}]$ are the membership and non-membership functions $,$ satisfying $0\le {\mu }_{C}\left(x\right),{\nu }_{C}\left(x\right),{\mu }_{C}\left(x\right)+{\nu }_{C}\left(x\right)\le 1.$ The degree of indeterminacy of $x$ to $C$ is defined as $\pi_{C} \left( x \right) = 1{-}\mu_{C} \left( x \right){-}\nu_{C} \left( x \right)$ The pair $\left({\mu }_{C},{\nu }_{C}\right)$ referred to as intuitionistic fuzzy number (IFN), in which ${\mu }_{c},{\nu }_{c}\in \left[\text{0,1}\right]$ and ${\mu }_{c}+{\nu }_{c}\le 1.$

Researchers have found that everyday decision-making situations are qualitative in nature and difficult to quantify. To tackle these aspects of refinement, Herrera and Martinez put forth the notion of linguistic variables as follows:

### Definition 3

(^36,37^). Let $S=\left\{{s}_{t}|t=\text{0,1},2,...,n\right\}$ be a finite set comprising linguistic terms possessing an odd cardinality, whereas ${s}_{n}$ illustrates the *n*th possible value of the linguistic variable. For instance, consider the linguistic variable “Height” with a corresponding set of three linguistic terms as $S=$*{s*_*0*_ = *Tall, s*_*1*_ = *Medium, s*_*2*_ = *Short}.*

Moreover, the set $S$ exhibits the following characteristics:

- Set $S$ is an ordered set:${s}_{q}\ge {s}_{r}\leftrightarrow q\ge r.$
- There exists negation, Max, Min operators as follows:
  i) $$Neg\left({s}_{q}\right)={s}_{r}$$ such that $$q=n-r$$
  ii) $$\mathit{max}\left({s}_{q},{s}_{r}\right)={s}_{q},\leftrightarrow q\ge r$$
  iii) $$\mathit{min}\left({s}_{q},{s}_{r}\right) ={s}_{q},\leftrightarrow q\le r$$

### Definition 4

(^43^). Let $S$ be a discrete linguistic term set. A continuous linguistic term set is defined as: $\overline{S}=\left\{{s}_{\alpha }|{s}_{0}\le {s}_{\alpha }\le {s}_{n},\alpha \in [0,n]\right\}$

where an element ${s}_{\alpha }\in S$ named as original linguistic term while an element ${s}_{\alpha }\notin S$ is referred to as a virtual linguistic term.

### Definition 5

(^43^). Given a continuous set of linguistic terms as $\overline{S}$ and a finite universal set $X.$ The representation of a LIFS $B$ is described as follows:

$$\text{B} = \left\{\left(x,{s}_{\mu }\left(x\right),{s}_{\nu }\left(x\right)\right)|x\in X\right\},$$

where ${s}_{\mu }\left(x\right),{s}_{\nu }\left(x\right)\in \overline{S}$ indicating the linguistic membership and linguistic non-membership degrees, respectively, of an element $x\in X.$ In addition, the condition $0\le \mu +\nu \le n$ must satisfy $x\in X.$ Moreover,$\pi \left(x\right)={s}_{n-\mu -\nu }$ represents the linguistic indeterminacy degree of $x$ to $B$. Specifically, if $\mu +\nu =n,$ then the LIFS attains its minimum linguistic indeterminacy degree, denoted as $\pi \left(x\right)={s}_{0}$.

The degrees of linguistic membership and non-membership of $x\in X$ are denoted by the notation $x=\left({s}_{\mu },{s}_{\nu }\right)$. This specific depiction of the element $x$ is referred to as a LIFN, where $0\le \mu +\nu \le n.$

### Definition 6

(^43^). Let ${g}_{1}=\left({s}_{\mu }, {s}_{\nu }\right)$ and ${g}_{2}=\left({s}_{\beta }, {s}_{\gamma }\right)$ be any two LIFNs; Then,

i) $${g}_{1}={g}_{2}$$ iff $${s}_{\upmu }={s}_{\upbeta }$$ and $${s}_{\upnu }= {s}_{\gamma }$$
ii) ${g}_{1}< {g}_{2}$ iff ${s}_{\upmu }<{s}_{\upbeta }$ and ${s}_{\upnu }>{s}_{\gamma }$
iii) $${g}_{1}^{c}=\left({s}_{\upnu } ,{s}_{\upmu }\right)$$
iv) $${g}_{1}\wedge {g}_{2}=\left(min\left({s}_{\upmu },{s}_{\upbeta }\right),max\left({s}_{\upnu },{s}_{\gamma }\right)\right)$$
V) $${g}_{1}\vee {g}_{2}=\left(\mathit{max}\left({s}_{\upmu },{s}_{\upbeta }\right),\mathit{min}\left({s}_{\upnu },{s}_{\gamma }\right)\right)$$

The subsequent operational laws are applicable to LIFNs when the t-norm and t-conorm are present.

### Definition 7

(^43^). Let ${g}_{1}=\left({s}_{\mu }, {s}_{\nu }\right)$ and ${g}_{2}=\left({s}_{\beta }, {s}_{\gamma }\right)$ be any two LIFNs and $\lambda >0$, Then;

- $${\text{i}) g}_{1}\oplus {g}_{2} =\left({{s}_{n(\mu /n + \beta /n - \mu \beta /{n}^{2}}}_{)},{{s}_{n(\nu \gamma /{n}^{2}}}_{)}\right)$$
- $${\text{ii}) g}_{1}\otimes {g}_{2} =\left({{s}_{n(\mu \beta /{n}^{2}}}_{)},{{s}_{n(\nu /n + \gamma /n - \nu \gamma /{n}^{2}}}_{)}\right)$$
- $$\lambda {g}_{1}=\left({{s}_{n(1-(1-\mu /n{)}^{\lambda }}}_{)},{s}_{n(\nu /n{)}^{\lambda }}\right)$$
- $${\text{iv}) g}_{1}^{\lambda }=\left({s}_{n(\mu /n{)}^{\lambda }},{s}_{n(1-(1-\nu /n{)}^{\lambda }}\right)$$

Fuzzy logic utilizes the fuzzy connectives t-norm and t-conorm, which were initially proposed by Dombi, to specify the union and intersection operations, respectively. In order to demonstrate the fusion of fuzzy sets, these connectives, which serve as mathematical representations of the logical conjunction (t-norm) and disjunction (t-conorm) of fuzzy propositions, are applied to the former.

### Definition 8

Ref^52^. The application of Dombi's t-norm and t-conorm to the arbitrary real integers $q$ and $r$ is delineated as follows:

i) $$Dom(q,r) =\frac{1}{1+{\left\{{\left(\frac{1-q}{q}\right)}^{m}+{\left(\frac{1-r}{r}\right)}^{m}\right\}}^\frac{1}{m}}$$
ii) $$Do{m}^{c}(q,r)=1-\frac{1}{1+{\left\{{\left(\frac{q}{1-q}\right)}^{m}+{\left(\frac{r}{1-r}\right)}^{m}\right\}}^\frac{1}{m}}$$

Here, $m\ge 1$ and $(q,r)\in {[\text{0,1}]}^{2}$. Moreover, (i) is referred to as the Dombi product and (ii) is referred to as Dombi sum.

## Formulation of novel score and accuracy functions for LIFNs

In this section, two novel accuracy and score functions for LIFNs pertaining to MADM problems are devised.

### Definition 9

Assume that $u=\left({s}_{\vartheta },{s}_{\delta }\right)$ is a LIFN with ${s}_{\vartheta },{s}_{\delta }\in \overline{S}=\left\{{s}_{\alpha }|{s}_{0}\le {s}_{\alpha }\le {s}_{n},\alpha \in [0,n]\right\}$. The score function $S$ for LIFN $u$ is formulated as follows:

$$S\left(u\right)={s}_{\left(\frac{n+\vartheta -\delta }{2+\vartheta }\right)}$$ where, $$0\le \frac{n+\vartheta -\delta }{2+\vartheta }\le n$$ .

The accuracy function is defined as.

$$\text{\rm M}\left(u\right)={s}_{\left(\frac{n-\left(\vartheta -\delta \right)}{2}\right)}$$ where,$$0\le \frac{n-\left(\vartheta -\delta \right)}{2}\le n$$

This definition assigns the following ranking criteria to any two LIFNs ${u}_{1}$ and ${u}_{2}$:

i. $S\left({u}_{1}\right)>S\left({u}_{2}\right)\Rightarrow {u}_{1}>{u}_{2}$ which means ${u}_{1}$ is stronger(larger) then ${u}_{2}$
ii. $S\left({u}_{1}\right)<S\left({u}_{2}\right)\Rightarrow {u}_{1}<{u}_{2}$ which means ${u}_{1}$ is weaker(smaller) then ${u}_{2}$
iii. $S\left({u}_{1}\right)=S\left({u}_{2}\right)\Rightarrow {u}_{1}\sim {u}_{2}$ which means ${u}_{1}$ and ${u}_{2}$ are incomparable

Then if,

- a) $${\rm M}\left({u}_{1}\right)>{\rm M}\left({u}_{2}\right)\Rightarrow {u}_{1}>{u}_{2}$$
- b) $${\rm M}\left({u}_{1}\right)<{\rm M}\left({u}_{2}\right)\Rightarrow {u}_{1}<{u}_{2}$$
- c) $${\rm M}({u}_{1})={\rm M}({u}_{2})\Rightarrow {u}_{1}\sim {u}_{2}$$

In order to showcase the efficacy of suggested scoring function for LIFNs, we provide the following example:

### Example 1.

Consider $k=\left({s}_{2},{s}_{3}\right)$ and $l=\left({s}_{1},{s}_{4}\right)$ be two LIFNs originated from a continuous linguistic term set $\overline{S}=\left\{{s}_{\alpha }|{s}_{0}\le {s}_{\alpha }\le {s}_{6},\alpha \in [\text{0,6}]\right\}$. Then by the application of definition 9, we get $S\left(k\right)={s}_{1.25}$ and $S\left(l\right)={s}_{1}$. Consequently, in view of definition 9 (i), we conclude that $k$ is stronger than $l.$ This observation indicates that $k$ is better than $l.$

## Dombi aggregation operators for LIFNs

In the subsequent section, we formulate the Dombi operations for LIF environment. In addition, we introduce two Dombi weighted aggregation operators, LIFDWA operator and LIFDWG operator, derived from the operational laws for LIFNs. We also investigate the essential characteristics of these operators.

### Definition 10

Consider any two LIFNs ${u}_{1}= ({s}_{{\mu }_{1}},{s}_{{\nu }_{1}})$ and ${u}_{2}= ({s}_{{\mu }_{2}},{s}_{{\nu }_{2}})$ defined on $\overline{S}=\left\{{s}_{\alpha }|{s}_{0}\le {s}_{\alpha }\le {s}_{n},\alpha \in \left[0,n\right]\right\}$ $\kappa \ge 1$ and $\lambda >0.$ The Dombi operations of t-norms and t-conorms for LIFNs are characterized as follows:

- $${\text{i})u}_{1}\oplus {u}_{2}=\left({s}_{n\left(1- \frac{1}{1+{\left\{{\left(\frac{{\mu }_{1}}{n-{\mu }_{1}}\right)}^{\kappa }+{\left(\frac{{\mu }_{2}}{n-{\mu }_{2}}\right)}^{\kappa }\right\}}^{\frac{1}{\kappa }}}\right)},{s}_{n\left(\frac{1}{1+{\left\{{\left(\frac{n-{\nu }_{1}}{{\nu }_{1}}\right)}^{\kappa }+{\left(\frac{n-{\nu }_{2}}{{\nu }_{2}}\right)}^{\kappa }\right\}}^{\frac{1}{\kappa }}}\right)}\right)$$
- $${\text{ii})u}_{1}\otimes {u}_{2}=\left({s}_{n\left(\frac{1}{1+{\left\{{\left(\frac{n-{\mu }_{1}}{{\mu }_{1}}\right)}^{\kappa }+{\left(\frac{n-{\mu }_{2}}{{\mu }_{2}}\right)}^{\kappa }\right\}}^{\frac{1}{\kappa }}}\right)},{s}_{n\left(1- \frac{1}{1+{\left\{{\left(\frac{{\nu }_{1}}{n-{\nu }_{1}}\right)}^{\kappa }+{\left(\frac{{\nu }_{2}}{n-{\nu }_{2}}\right)}^{\kappa }\right\}}^{\frac{1}{\kappa }}}\right)}\right)$$
- $$\lambda {u}_{1}=\left({s}_{n\left(1- \frac{1}{1+{\left\{\lambda {\left(\frac{{\mu }_{1}}{n-{\mu }_{1}}\right)}^{\kappa }\right\}}^{\frac{1}{\kappa }}}\right)},{s}_{n\left(\frac{1}{1+{\left\{\lambda {\left(\frac{n-{\nu }_{1}}{{\nu }_{1}}\right)}^{\kappa }\right\}}^{\frac{1}{\kappa }}}\right)}\right)$$
- $${{\text{iv})u}_{1}}^{\lambda }=\left({s}_{n\left(\frac{1}{1+{\left\{\lambda {\left(\frac{n-{\mu }_{1}}{{\mu }_{1}}\right)}^{\kappa }\right\}}^{\frac{1}{\kappa }}}\right)},{s}_{n\left(1-\frac{1}{1+{\left\{\lambda {\left(\frac{{\nu }_{1}}{n-{\nu }_{1}}\right)}^{\kappa }\right\}}^{\frac{1}{\kappa }}}\right)}\right)$$

### Fundamental characteristics of LIFDWA operator

In the following segment, we define LIFDWA operator and investigate its fundamental aspects.

#### Definition 11

Consider a collection $\Omega$ consisting of $q$ number of LIFNs, ${g}_{i}=({s}_{{\vartheta }_{i}},{s}_{{\delta }_{i}})$,$\left(i=\text{1,2},\dots ,q\right)$ defined on $\overline{S}=\left\{{s}_{\alpha }|{s}_{0}\le {s}_{\alpha }\le {s}_{n},\alpha \in \left[0,n\right]\right\}$ and $\varphi =({\varphi }_{1},{\varphi }_{2},...,{\varphi }_{q}{)}^{T}$ is the associated weight vector of these LIFNs ${g}_{i}$ where $0\le {\varphi }_{i}\le 1$, such that ${\sum }_{i=1}^{q}{\varphi }_{i}=1$ and the parameter $\kappa \ge 1.$ Then, The LIFDWA operator is characterized by a function LIFDWA: ${\Omega }^{q}\to \Omega$ defined by the rule;

$$LIFDW{A}_{\varphi }\left({g}_{1},{g}_{2},\dots ,{g}_{q}\right)={\varphi }_{1}{g}_{1}\oplus {\varphi }_{2}{g}_{2}\oplus {\varphi }_{3}{g}_{3}\oplus ...\oplus {\varphi }_{q}{g}_{q}$$

$${\oplus }_{i=1}^{q}{\varphi }_{i}{g}_{i}=\left({s}_{n\left(1- \frac{1}{1+{\left\{{\sum }_{i=1}^{q}{\varphi }_{i}{\left(\frac{{\vartheta }_{i}}{n-{\vartheta }_{i}}\right)}^{\kappa }\right\}}^{\frac{1}{\kappa }}}\right)},{s}_{n\left(\frac{1}{1+{\left\{{\sum }_{i=1}^{q}{\varphi }_{i}{\left(\frac{n-{\delta }_{i}}{{\delta }_{i}}\right)}^{\kappa }\right\}}^{\frac{1}{\kappa }}}\right)}\right)$$

#### Theorem 1.

*Consider* $q$ number of LIFNs indicated as ${g}_{i}= \left({s}_{{\vartheta }_{i}},{s}_{{\delta }_{i}}\right),$ $i=\left(\text{1,2},...,q\right)$ defined on $\overline{S}=\left\{{s}_{\alpha }|\alpha \in \left[0,n\right]\right\}$ and $\varphi ={\left({\varphi }_{1},{\varphi }_{2},...,{\varphi }_{q}\right)}^{T}$ is the associated weight vector of ${g}_{i}$ with ${\varphi }_{i}>0$ and ${\sum }_{i=1}^{q}{\varphi }_{i}=1$. The aggregated value of LIFNs ${g}_{i}$ acquired within LIFDWA operator is a LIFN and is formulated as: $LIFDW{A}_{\varphi }\left({g}_{1},{g}_{2},...,{g}_{q}\right)={\oplus }_{i=1}^{q}{\varphi }_{i}{g}_{i}$

$${\oplus }_{i=1}^{q}{\varphi }_{i}{g}_{i}=\left({s}_{n\left(1- \frac{1}{1+{\left\{{\sum }_{i=1}^{q}{\varphi }_{i}{\left(\frac{{\vartheta }_{i}}{n-{\vartheta }_{i}}\right)}^{\kappa }\right\}}^{\frac{1}{\kappa }}}\right)},{s}_{n\left(\frac{1}{1+{\left\{{\sum }_{i=1}^{q}{\varphi }_{i}{\left(\frac{n-{\delta }_{i}}{{\delta }_{i}}\right)}^{\kappa }\right\}}^{\frac{1}{\kappa }}}\right)}\right)$$

#### Proof.

We demonstrate the validity of this assertion by employing mathematical induction. Suppose that $q=2,$ then, we have ${g}_{1}=\left({s}_{{\vartheta }_{1}},{s}_{{\delta }_{1}}\right)$ and ${g}_{2}=\left({s}_{{\vartheta }_{2}},{s}_{{\delta }_{2}}\right).$

Then, by applying the Dombi operations designed for LIFNs in definition 10, we acquire the following expressions

$${\varphi }_{1}{g}_{1}=\left({s}_{n\left(1- \frac{1}{1+{\left\{{\varphi }_{1}{\left(\frac{{\vartheta }_{1}}{n-{\vartheta }_{1}}\right)}^{k}\right\}}^\frac{1}{k}}\right)},{s}_{n\left(\frac{1}{1+{\left\{{\varphi }_{1}{\left(\frac{n-{\delta }_{1}}{{\delta }_{1}}\right)}^{k}\right\}}^\frac{1}{k}}\right)}\right)$$ and $${\varphi }_{2}{g}_{2}=\left({s}_{n\left(1- \frac{1}{1+{\left\{{\varphi }_{2}{\left(\frac{{\vartheta }_{2}}{n-{\vartheta }_{2}}\right)}^{k}\right\}}^\frac{1}{k}}\right)},{s}_{n\left(\frac{1}{1+{\left\{{\varphi }_{2}{\left(\frac{n-{\delta }_{2}}{{\delta }_{2}}\right)}^{k}\right\}}^\frac{1}{k}}\right)}\right)$$

The application of definition 11 to the above relations yield the following outcome:

$$LIFDW{A}_{\varphi }\left({g}_{1},{g}_{2}\right)={\varphi }_{1}{g}_{1}\oplus {\varphi }_{2}{g}_{2}$$

$$=\left({s}_{n\left(1- \frac{1}{1+{\left\{{\varphi }_{1}{\left(\frac{{\vartheta }_{1}}{n-{\vartheta }_{1}}\right)}^{k}\right\}}^\frac{1}{k}}\right)},{s}_{n\left(\frac{1}{1+{\left\{{\varphi }_{1}{\left(\frac{n-{\delta }_{1}}{{\delta }_{1}}\right)}^{k}\right\}}^\frac{1}{k}}\right)}\right) \oplus \left({s}_{n\left(1- \frac{1}{1+{\left\{{\varphi }_{2}{\left(\frac{{\vartheta }_{2}}{n-{\vartheta }_{2}}\right)}^{k}\right\}}^\frac{1}{k}}\right)},{s}_{n\left(\frac{1}{1+{\left\{{\varphi }_{2}{\left(\frac{n-{\delta }_{2}}{{\delta }_{2}}\right)}^{k}\right\}}^\frac{1}{k}}\right)}\right)$$

It follows that:

$${\varphi }_{1}{g}_{1}\oplus {\varphi }_{2}{g}_{2}=\left({s}_{n\left(1- \frac{1}{1+{\left\{{\varphi }_{1}{\left(\frac{{\vartheta }_{1}}{n-{\vartheta }_{1}}\right)}^{k}+{\varphi }_{2}{\left(\frac{{\vartheta }_{2}}{n-{\vartheta }_{2}}\right)}^{k}\right\}}^\frac{1}{k}}\right)},{s}_{n\left(\frac{1}{1+{\left\{{\varphi }_{1}{\left(\frac{n-{\delta }_{1}}{{\delta }_{1}}\right)}^{k}+{\varphi }_{2}{\left(\frac{n-{\delta }_{2}}{{\delta }_{2}}\right)}^{k}\right\}}^\frac{1}{k}}\right)}\right)$$

Consequently,

$$LIFDW{A}_{\varphi }\left({g}_{1},{g}_{2}\right)=\left({s}_{n\left(1- \frac{1}{1+{\left\{{\sum }_{i=1}^{2}{\varphi }_{i}{\left(\frac{{\vartheta }_{i}}{n-{\vartheta }_{i}}\right)}^{k}\right\}}^\frac{1}{k}}\right)},{s}_{n\left(\frac{1}{1+{\left\{{{\sum }_{i=1}^{2}{\varphi }_{i}\left(\frac{n-{\delta }_{i}}{{\delta }_{i}}\right)}^{k}\right\}}^\frac{1}{k}}\right)}\right)$$

Hence, the assertion holds true when $q$ is equal to $2$.

Assume that the theorem is valid when $q$ is equal to $p$. Then,

$$LIFDW{A}_{\varphi }\left({g}_{1},{g}_{2},\dots ,{g}_{p}\right)={\oplus }_{i=1}^{p}\left({\varphi }_{i}{g}_{i}\right)=\left({s}_{n\left(1- \frac{1}{1+{\left\{{\sum }_{i=1}^{p}{\varphi }_{i}{\left(\frac{{\vartheta }_{i}}{n-{\vartheta }_{i}}\right)}^{\kappa }\right\}}^{\frac{1}{\kappa }}}\right)},{s}_{n\left(\frac{1}{1+{\left\{{\sum }_{i=1}^{p}{\varphi }_{i}{\left(\frac{n-{\delta }_{i}}{{\delta }_{i}}\right)}^{\kappa }\right\}}^{\frac{1}{\kappa }}}\right)}\right).$$

Now, consider the case for $q=p+1,$ then.

$$LIFDW{A}_{\varphi }\left({g}_{1},{g}_{2},...,{g}_{p},{g}_{p+1}\right)={\oplus }_{i=1}^{p}\left({\varphi }_{i}{g}_{i}\right)\oplus \left({\varphi }_{p+1}{g}_{p+1}\right)=\left({s}_{n\left(1- \frac{1}{1+{\left\{{\sum }_{i=1}^{p}{\varphi }_{i}{\left(\frac{{\vartheta }_{i}}{n-{\vartheta }_{i}}\right)}^{\kappa }\right\}}^{\frac{1}{\kappa }}}\right)},{s}_{n\left(\frac{1}{1+{\left\{{\sum }_{i=1}^{p}{\varphi }_{i}{\left(\frac{n-{\delta }_{i}}{{\delta }_{i}}\right)}^{\kappa }\right\}}^{\frac{1}{\kappa }}}\right)}\right)\oplus \left({s}_{n\left(1- \frac{1}{1+{\left\{{\varphi }_{p+1}{\left(\frac{{\vartheta }_{p+1}}{n-{\vartheta }_{p+1}}\right)}^{\kappa }\right\}}^{\frac{1}{\kappa }}}\right)},{s}_{n\left(\frac{1}{1+{\left\{{\varphi }_{p+1}{\left(\frac{n-{\delta }_{p+1}}{{\delta }_{p+1}}\right)}^{\kappa }\right\}}^{\frac{1}{\kappa }}}\right)}\right)=\left({s}_{n\left(1- \frac{1}{1+{\left\{{\sum }_{i=1}^{p+1}{\varphi }_{i}{\left(\frac{{\vartheta }_{i}}{n-{\vartheta }_{i}}\right)}^{\kappa }\right\}}^{\frac{1}{\kappa }}}\right)},{s}_{n\left(\frac{1}{1+{\left\{{\sum }_{i=1}^{p+1}{\varphi }_{i}{\left(\frac{n-{\delta }_{i}}{{\delta }_{i}}\right)}^{\kappa }\right\}}^{\frac{1}{\kappa }}}\right)}\right).$$

The outcome is therefore valid for $q=p+1.$ Thus, the aforementioned procedure illustrates the reality that every positive integral value of $q$ yields the same result.

#### Example 2.

Consider three clients to rate a hotel’s services. The opinion of three clients is summarized in the form of LIFNs, ${g}_{1}=\left({s}_{2.367},{s}_{3}\right),{g}_{2}=\left({s}_{2.841},{s}_{1.335}\right)$ and ${g}_{3}=\left({s}_{3.026},{s}_{2.195}\right)$ defined on a continuous linguistic term set $\overline{S}=\left\{{s}_{\alpha }|{s}_{0}\le {s}_{\alpha }\le {s}_{6},\alpha \in [\text{0,6}]\right\}$ with the corresponding weight vectors of three clients $\varphi ={\left(\text{0.3,0.2,0.5}\right)}^{T}$ and parameter $\kappa =3,$ the LIFDWA operator can be used to obtain the aggregated value of the three LIFNs as:

$$LIFDW{A}_{\varphi }\left({g}_{1},{g}_{2},{g}_{3}\right)={\oplus }_{i=1}^{3}{\varphi }_{i}{g}_{i}$$

$$=\left({s}_{n\left(1- \frac{1}{1+{\left\{{\sum }_{i=1}^{3}{\varphi }_{i}{\left(\frac{{\vartheta }_{i}}{n-{\vartheta }_{i}}\right)}^{\kappa }\right\}}^{\frac{1}{\kappa }}}\right)},{s}_{n\left(\frac{1}{1+{\left\{{{\sum }_{i=1}^{3}{\varphi }_{i}\left(\frac{n-{\delta }_{i}}{{\delta }_{i}}\right)}^{\kappa }\right\}}^{\frac{1}{\kappa }}}\right)}\right)$$

Consequently, $$LIFDW{A}_{\varphi }\left({g}_{1},{g}_{2},{g}_{3}\right)=\left({s}_{2.8597},{s}_{1.8444}\right)$$.

Hence, the above discussion illustrates the validity of the fact described in theorem 1.

Any number of LIFNs can have the idempotency feature in the LIFDWA operator setup. Here’s how this case is justified:

#### Theorem 2.

*(Idempotency):* Consider $q$ number of LIFNs indicated as ${g}_{i}= \left({s}_{{\vartheta }_{i}},{s}_{{\delta }_{i}}\right),$ $i=\left(\text{1,2},...,q\right)$ defined on $\overline{S}=\left\{{s}_{\alpha }|\alpha \in \left[0,n\right]\right\}$ and $\varphi ={\left({\varphi }_{1},{\varphi }_{2},...,{\varphi }_{q}\right)}^{T}$ is the associated weight vector of ${g}_{i}$ with ${\varphi }_{i}>0$ and ${\sum }_{i=1}^{q}{\varphi }_{i}=1$. If all ${g}_{i}=\left({s}_{{\vartheta }_{i}},{s}_{{\delta }_{i}}\right)=\left({s}_{\vartheta },{s}_{\delta }\right)$ for any $i.$ Then; $LIFDW{A}_{\varphi }\left({g}_{1},{g}_{2},...,{g}_{q}\right)=\left({s}_{\vartheta },{s}_{\delta }\right)$.

#### Proof

Given that ${g}_{i}=g=\left({s}_{\theta },{s}_{\sigma }\right)$ for any $i.$ Then, by definition 11, this implies that

$$LIFDW{A}_{\varphi }\left({g}_{1},{g}_{2},...,{g}_{q}\right)={\oplus }_{i=1}^{q}\left({\varphi }_{i}{g}_{i}\right)$$

$$=\left({s}_{n\left(1- \frac{1}{1+{\left\{{\sum }_{i=1}^{q}{\varphi }_{i}{\left(\frac{{\vartheta }_{i}}{n-{\vartheta }_{i}}\right)}^{\kappa }\right\}}^{\frac{1}{\kappa }}}\right)},{s}_{n\left(\frac{1}{1+{\left\{{\sum }_{i=1}^{q}{\varphi }_{i}{\left(\frac{n-{\delta }_{i}}{{\delta }_{i}}\right)}^{\kappa }\right\}}^{\frac{1}{\kappa }}}\right)}\right)$$

$$=\left({s}_{n\left(1- \frac{1}{1+\left(\frac{\vartheta }{n-\vartheta }\right){\left\{{\sum }_{i=1}^{q}{\varphi }_{i}\right\}}^{\frac{1}{\kappa }}}\right)},{s}_{n\left(\frac{1}{1+\left(\frac{n-\delta }{\delta }\right){\left\{{\sum }_{i=1}^{q}{\varphi }_{i}\right\}}^{\frac{1}{\kappa }}}\right)}\right)$$

$$=\left({s}_{n\left(1- \frac{1}{1+\left\{\left(\frac{\vartheta }{n-\vartheta }\right)\right\}}\right)},{s}_{n\left(\frac{1}{1+\left\{\left(\frac{n-\delta }{\delta }\right)\right\}}\right)}\right)$$

$$=\left({s}_{\vartheta },{s}_{\delta }\right).$$

Thus, $$LIFDW{A}_{\varphi }\left({g}_{1},{g}_{2},...,{g}_{q}\right)=g$$.

#### Theorem 3

*(Boundedness)*: Consider $q$ number of LIFNs indicated as ${g}_{i}= \left({s}_{{\vartheta }_{i}},{s}_{{\delta }_{i}}\right),$ $i=\left(\text{1,2},...,q\right)$ defined on $\overline{S}=\left\{{s}_{\alpha }|\alpha \in \left[0,n\right]\right\}$ and $\varphi ={\left({\varphi }_{1},{\varphi }_{2},...,{\varphi }_{q}\right)}^{T}$ is the associated weight vector of ${g}_{i}$ with ${\varphi }_{i}>0$ and ${\sum }_{i=1}^{q}{\varphi }_{i}=1.$ Assume ${g}^{-}=\mathit{min}\left\{{g}_{1},{g}_{2},...,{g}_{q}\right\}$ and ${g}^{+}=\mathit{max}\left\{{g}_{1},{g}_{2},...,{g}_{q}\right\}$.Then ${g}^{-}\le LIFDW{A}_{\varphi }\left({g}_{1},{g}_{2},...,{g}_{q}\right)\le {g}^{+}.$

#### Proof.

Consider the outcome that is accomplished by applying the LIFDWA operator to the collection of LIFNs as:${LIFDWA}_{\varphi }\left({g}_{1},{g}_{2},...,{g}_{q}\right)=\left({s}_{\vartheta },{s}_{\delta }\right).$

Suppose that, ${g}^{-}=\mathit{min}\left\{{g}_{1},{g}_{2},...,{g}_{q}\right\}=\left({s}_{{\vartheta }^{-}},{s}_{{\delta }^{-}}\right)$ and ${g}^{+}=\mathit{max}\left\{{g}_{1},{g}_{2},...,{g}_{q}\right\}=\left({s}_{{\vartheta }^{+}},{s}_{{\delta }^{+}}\right)$ where $${s}_{{\vartheta }^{-}}=\begin{array}{c}min\\ i\end{array}\left\{{s}_{{\vartheta }_{i}}\right\}$$ , $${s}_{{\delta }^{-}}=\begin{array}{c}max\\ i\end{array}\left\{{s}_{{\delta }_{i}}\right\}$$ and $${s}_{{\vartheta }^{+}}=\begin{array}{c}max\\ i\end{array}\left\{{s}_{{\vartheta }_{i}}\right\} , {s}_{{\delta }^{+}}=\begin{array}{c}min\\ i\end{array}\left\{{s}_{{\delta }_{i}}\right\}$$.

Since, For each LIFN ${g}_{i},$ $$\begin{array}{c}min\\ i\end{array}\left\{{s}_{{\vartheta }_{i}}\right\}\le {s}_{{\vartheta }_{i}}\le \begin{array}{c}max\\ i\end{array}\left\{{s}_{{\vartheta }_{i}}\right\}$$

$$\Rightarrow \begin{array}{c}min\\ i\end{array}\left\{{s}_{{\left(\frac{{\vartheta }_{i}}{n-{\vartheta }_{i}}\right)}^{\kappa }}\right\}\le {s}_{{\left(\frac{{\vartheta }_{i}}{n-{\vartheta }_{i}}\right)}^{\kappa }}\le \begin{array}{c}max\\ i\end{array}\left\{{s}_{{\left(\frac{{\vartheta }_{i}}{n-{\vartheta }_{i}}\right)}^{\kappa }}\right\}$$

$$\Rightarrow \begin{array}{c}min\\ i\end{array}\left\{{s}_{{\left\{{{\sum }_{i=1}^{q}{\varphi }_{i}\left(\frac{{\vartheta }_{i}}{n-{\vartheta }_{i}}\right)}^{\kappa }\right\}}^\frac{1}{k}}\right\}\le \left({s}_{{\left\{{{\sum }_{i=1}^{q}{\varphi }_{i}\left(\frac{{\vartheta }_{i}}{n-{\vartheta }_{i}}\right)}^{\kappa }\right\}}^\frac{1}{k}}\right)\le \begin{array}{c}max\\ i\end{array}\left\{{s}_{{\left\{{{\sum }_{i=1}^{q}{\varphi }_{i}\left(\frac{{\vartheta }_{i}}{n-{\vartheta }_{i}}\right)}^{\kappa }\right\}}^\frac{1}{k}}\right\}$$

$$\Rightarrow \begin{array}{c}max\\ i\end{array}\left\{{s}_{\left(\frac{1}{1+{\left\{{{\sum }_{i=1}^{q}{\varphi }_{i}\left(\frac{{\vartheta }_{i}}{n-{\vartheta }_{i}}\right)}^{\kappa }\right\}}^\frac{1}{k}}\right)}\right\}\le {s}_{\left(\frac{1}{1+{\left\{{{\sum }_{i=1}^{q}{\varphi }_{i}\left(\frac{{\vartheta }_{i}}{n-{\vartheta }_{i}}\right)}^{\kappa }\right\}}^\frac{1}{k}}\right)}\le \begin{array}{c}min\\ i\end{array}\left\{{s}_{\left(\frac{1}{1+{\left\{{{\sum }_{i=1}^{q}{\varphi }_{i}\left(\frac{{\vartheta }_{i}}{n-{\vartheta }_{i}}\right)}^{\kappa }\right\}}^\frac{1}{k}}\right)}\right\}$$

$$\Rightarrow \begin{array}{c}min\\ i\end{array}\left\{{s}_{\left(1- \frac{1}{1+{\left\{{{\sum }_{i=1}^{q}{\varphi }_{i}\left(\frac{{\vartheta }_{i}}{n-{\vartheta }_{i}}\right)}^{\kappa }\right\}}^\frac{1}{k}}\right)}\right\}\le {s}_{\left(1- \frac{1}{1+{\left\{{{\sum }_{i=1}^{q}{\varphi }_{i}\left(\frac{{\vartheta }_{i}}{n-{\vartheta }_{i}}\right)}^{\kappa }\right\}}^\frac{1}{k}}\right)}\le \begin{array}{c}max\\ i\end{array}\left\{{s}_{\left(1- \frac{1}{1+{\left\{{{\sum }_{i=1}^{q}{\varphi }_{i}\left(\frac{{\vartheta }_{i}}{n-{\vartheta }_{i}}\right)}^{\kappa }\right\}}^\frac{1}{k}}\right)}\right\}$$

$$\Rightarrow {s}_{n\left(1- \frac{1}{1+{\left\{{\sum }_{i=1}^{q}{\varphi }_{i}{\left(\frac{{\vartheta }^{\_}}{n-{\vartheta }^{-}}\right)}^{\kappa }\right\}}^{\frac{1}{\kappa }}}\right)}\le {s}_{n\left(1- \frac{1}{1+{\left\{{\sum }_{i=1}^{q}{\varphi }_{i}{\left(\frac{{\vartheta }_{i}}{n-{\vartheta }_{i}}\right)}^{\kappa }\right\}}^{\frac{1}{\kappa }}}\right)}\le {s}_{n\left(1- \frac{1}{1+{\left\{{\sum }_{i=1}^{q}{\varphi }_{i}{\left(\frac{{\vartheta }^{+}}{n-{\vartheta }^{+}}\right)}^{\kappa }\right\}}^{\frac{1}{\kappa }}}\right)}$$

1 $$\Rightarrow {s}_{{\vartheta }^{-}}\le {s}_{{\vartheta }_{i}}\le {s}_{{\vartheta }^{+}}$$

Furthermore, by implementing the aforementioned mathematical approach for the relation $$\begin{array}{c}max\\ i\end{array}\left\{{s}_{{\delta }_{i}}\right\}\le {s}_{{\delta }_{i}}\le \begin{array}{c}min\\ i\end{array}\left\{{s}_{{\delta }_{i}}\right\},$$ we obtain the following outcome:

2 $${s}_{{\delta }^{-}}\le {s}_{{\delta }_{i}}\le {s}_{{\delta }^{+}}$$

The subsequent relationship is derived through a comparison of (1) and (2).

$${g}^{-}\le LIFDW{A}_{\varphi }\left({g}_{1},{g}_{2},...,{g}_{q}\right)\le {g}^{+}$$

#### Theorem 4.

*(Monotonicity):* Consider $q$ number of LIFNs indicated as ${g}_{i}= ({s}_{{\vartheta }_{i}},{s}_{{\delta }_{i}})$ and ${{g}_{i}}^{*}= \left({{s}_{{\vartheta }_{i}}}^{*},{{s}_{{\delta }_{i}}}^{*}\right),$ $i=\left(\text{1,2},...,q\right)$ defined on $\overline{S}=\left\{{s}_{\alpha }|\alpha \in \left[0,n\right]\right\}$ and $\varphi ={\left({\varphi }_{1},{\varphi }_{2},...,{\varphi }_{q}\right)}^{T}$ is the associated weight vector of ${g}_{i}$ with ${\varphi }_{i}>0$ and ${\sum }_{i=1}^{q}{\varphi }_{i}=1$. If ${s}_{{\vartheta }_{i}}\le {{s}_{{\vartheta }_{i}}}^{*}$ and ${s}_{{\delta }_{i}}\ge {{s}_{{\delta }_{i}}}^{*}.$ Then, ${LIFDWA}_{\varphi }\left({g}_{1},{g}_{2},\dots ,{g}_{q}\right)\le {LIFDWA}_{\varphi }\left({g}_{1}^{*},{g}_{2}^{*},\dots ,{g}_{q}^{*}\right)$

#### Proof.

Consider ${s}_{{\vartheta }_{i}}\le {{s}_{{\vartheta }_{i}}}^{*}$ for all $i,$ then

$${s}_{{\left(\frac{{\vartheta }_{i}}{n-{\vartheta }_{i}}\right)}^{\kappa }}\le {s}_{{\left(\frac{{{\vartheta }_{i}}^{*}}{n-{{\vartheta }_{i}}^{*}}\right)}^{\kappa }}$$

$$\Rightarrow {s}_{{\left\{{\sum }_{i=1}^{q}{\varphi }_{i}{\left(\frac{{\vartheta }_{i}}{n-{\vartheta }_{i}}\right)}^{\kappa }\right\}}^{\frac{1}{\kappa }}}\le {s}_{{\left\{{\sum }_{i=1}^{q}{\varphi }_{i}{\left(\frac{{{\vartheta }_{i}}^{*}}{n-{{\vartheta }_{i}}^{*}}\right)}^{\kappa }\right\}}^{\frac{1}{\kappa }}}$$

$$\Rightarrow {s}_{\left(1+{\left\{{\sum }_{i=1}^{q}{\varphi }_{i}{\left(\frac{{\vartheta }_{i}}{n-{\vartheta }_{i}}\right)}^{\kappa }\right\}}^{\frac{1}{\kappa }}\right)}\le {s}_{\left(1+{\left\{{\sum }_{i=1}^{q}{\varphi }_{i}{\left(\frac{{{\vartheta }_{i}}^{*}}{n-{{\vartheta }_{i}}^{*}}\right)}^{\kappa }\right\}}^{\frac{1}{\kappa }}\right)}$$

$$\Rightarrow {s}_{\left(\frac{1}{1+{\left\{{\sum }_{i=1}^{q}{\varphi }_{i}{\left(\frac{{\vartheta }_{i}}{n-{\vartheta }_{i}}\right)}^{\kappa }\right\}}^{\frac{1}{\kappa }}}\right)}\ge {s}_{\left(\frac{1}{1+{\left\{{\sum }_{i=1}^{q}{\varphi }_{i}{\left(\frac{{{\vartheta }_{i}}^{*}}{n-{{\vartheta }_{i}}^{*}}\right)}^{\kappa }\right\}}^{\frac{1}{\kappa }}}\right)}$$

$$\Rightarrow {s}_{\left(1- \frac{1}{1+{\left\{{\sum }_{i=1}^{q}{\varphi }_{i}{\left(\frac{{\vartheta }_{i}}{n-{\vartheta }_{i}}\right)}^{\kappa }\right\}}^{\frac{1}{\kappa }}}\right)}\le {s}_{\left(1- \frac{1}{1+{\left\{{\sum }_{i=1}^{q}{\varphi }_{i}{\left(\frac{{{\vartheta }_{i}}^{*}}{n-{{\vartheta }_{i}}^{*}}\right)}^{\kappa }\right\}}^{\frac{1}{\kappa }}}\right)}$$

3 $$\Rightarrow {s}_{n\left(1- \frac{1}{1+{\left\{{\sum }_{i=1}^{q}{\varphi }_{i}{\left(\frac{{\vartheta }_{i}}{n-{\vartheta }_{i}}\right)}^{\kappa }\right\}}^{\frac{1}{\kappa }}}\right)}\le {s}_{n\left(1- \frac{1}{1+{\left\{{\sum }_{i=1}^{q}{\varphi }_{i}{\left(\frac{{{\vartheta }_{i}}^{*}}{n-{{\vartheta }_{i}}^{*}}\right)}^{\kappa }\right\}}^{\frac{1}{\kappa }}}\right)}.$$

Moreover, ${s}_{{\delta }_{i}}\ge {{s}_{{\delta }_{i}}}^{*}\forall i,$ However, on the same lines we can demonstrate that

4 $${s}_{n\left(\frac{1}{1+{\left\{{\sum }_{i=1}^{q}{\varphi }_{i}{\left(\frac{n-{\delta }_{i}}{{\delta }_{i}}\right)}^{\kappa }\right\}}^{\frac{1}{\kappa }}}\right)}\ge {s}_{n\left(\frac{1}{1+{\left\{{\sum }_{i=1}^{q}{\varphi }_{i}{\left(\frac{n-{{\delta }_{i}}^{*}}{{{\delta }_{i}}^{*}}\right)}^{\kappa }\right\}}^{\frac{1}{\kappa }}}\right)}$$

By comparing (3), (4) and using definition 6, we have ${LIFDWA}_{\varphi }\left({g}_{1},{g}_{2},\dots ,{g}_{q}\right)\le {LIFDWA}_{\varphi }\left({g}_{1}^{*},{g}_{2}^{*},\dots ,{g}_{q}^{*}\right)$.

### Fundamental characteristics of LIFDWG operator

The subsequent section provides a definition of the LIFDWG operator and examines its fundamental characteristics.

#### Definition 12:

Consider a collection $\Omega$ consisting of $q$ number of LIFNs, ${g}_{i}=({s}_{{\vartheta }_{i}},{s}_{{\delta }_{i}})$,$\left(i=\text{1,2},\dots ,q\right)$ defined on $\overline{S}=\left\{{s}_{\alpha }|{s}_{0}\le {s}_{\alpha }\le {s}_{n},\alpha \in \left[0,n\right]\right\}$ and $\varphi =({\varphi }_{1},{\varphi }_{2},...,{\varphi }_{q}{)}^{T}$ is the associated weight vector of these LIFNs ${g}_{i}$ where $0\le {\varphi }_{i}\le 1$, such that ${\sum }_{i=1}^{q}{\varphi }_{i}=1$ and the parameter $\kappa \ge 1.$ Then, The LIFDWG operator is characterized by a function LIFDWG: ${\Omega }^{q}\to \Omega$ defined by the rule;

$$LIFDW{G}_{\varphi }\left({g}_{1},{g}_{2},\dots ,{g}_{q}\right)={{g}_{1}}^{{\varphi }_{1}}\otimes {{g}_{2}}^{{\varphi }_{2}}\otimes ...\otimes {{g}_{q}}^{{\varphi }_{q}}$$

$${\otimes }_{i=1}^{q}{{g}_{i}}^{{\varphi }_{i}}=\left({s}_{n\left(\frac{1}{1+{\left\{{\sum }_{i=1}^{q}{\varphi }_{i}{\left(\frac{n-{\vartheta }_{i}}{{\vartheta }_{i}}\right)}^{\kappa }\right\}}^{\frac{1}{\kappa }}}\right)},{s}_{n\left(1- \frac{1}{1+{\left\{{\sum }_{i=1}^{q}{\varphi }_{i}{\left(\frac{{\delta }_{i}}{n-{\delta }_{i}}\right)}^{\kappa }\right\}}^{\frac{1}{\kappa }}}\right)}\right)$$

#### Theorem 5.

Consider $q$ number of LIFNs indicated as ${g}_{i}= \left({s}_{{\vartheta }_{i}},{s}_{{\delta }_{i}}\right),$ $i=\left(\text{1,2},...,q\right)$ defined on $\overline{S}=\left\{{s}_{\alpha }|\alpha \in \left[0,n\right]\right\}$ and $\varphi ={\left({\varphi }_{1},{\varphi }_{2},...,{\varphi }_{q}\right)}^{T}$ is the associated weight vector of ${g}_{i}$ with ${\varphi }_{i}>0$ and ${\sum }_{i=1}^{q}{\varphi }_{i}=1$. The aggregated value of LIFNs ${g}_{i}$ acquired within LIFDWG operator is a LIFN and is expressed in the following fashion:

$$LIFDW{G}_{\varphi }\left({g}_{1},{g}_{2},...,{g}_{q}\right)={\otimes }_{i=1}^{q}{{g}_{i}}^{{\varphi }_{i}}$$

$${\otimes }_{i=1}^{q}{{g}_{i}}^{{\varphi }_{i}}=\left({s}_{n\left(\frac{1}{1+{\left\{{\sum }_{i=1}^{q}{\varphi }_{i}{\left(\frac{n-{\vartheta }_{i}}{{\vartheta }_{i}}\right)}^{\kappa }\right\}}^{\frac{1}{\kappa }}}\right)},{s}_{n\left(1- \frac{1}{1+{\left\{{\sum }_{i=1}^{q}{\varphi }_{i}{\left(\frac{{\delta }_{i}}{n-{\delta }_{i}}\right)}^{\kappa }\right\}}^{\frac{1}{\kappa }}}\right)}\right)$$

#### Proof.

We demonstrate the validity of this assertion by employing mathematical induction. Suppose that $q=2,$ then, we have ${g}_{1}=\left({s}_{{\vartheta }_{1}},{s}_{{\delta }_{1}}\right)$ and ${g}_{2}=\left({s}_{{\vartheta }_{2}},{s}_{{\delta }_{2}}\right)$.

We get the following equations by using LIFN-specific Dombi operations:

$${{g}_{1}}^{{\varphi }_{1}}=\left({s}_{n\left(\frac{1}{1+{\left\{{\varphi }_{1}{\left(\frac{n-{\vartheta }_{1}}{{\vartheta }_{1}}\right)}^{k}\right\}}^\frac{1}{k}}\right)},{s}_{n\left(1- \frac{1}{1+{\left\{{\varphi }_{1}{\left(\frac{{\delta }_{1}}{n-{\delta }_{1}}\right)}^{k}\right\}}^\frac{1}{k}}\right)}\right)$$

$${{g}_{2}}^{{\varphi }_{2}}=\left({s}_{n\left(\frac{1}{1+{\left\{{\varphi }_{2}{\left(\frac{n-{\vartheta }_{2}}{{\vartheta }_{2}}\right)}^{k}\right\}}^\frac{1}{k}}\right)},{s}_{n\left(1- \frac{1}{1+{\left\{{\varphi }_{2}{\left(\frac{{\delta }_{2}}{n-{\delta }_{2}}\right)}^{k}\right\}}^\frac{1}{k}}\right)}\right)$$

Applying definition 12 to the aforementioned relations results in $LIFDW{G}_{\varphi }\left({g}_{1},{g}_{2}\right)={{g}_{1}}^{{\varphi }_{1}}\otimes {{g}_{2}}^{{\varphi }_{2}}$. So,

$${{g}_{1}}^{{\varphi }_{1}}\otimes {{g}_{2}}^{{\varphi }_{2}}=\left({s}_{n\left(\frac{1}{1+{\left\{{\varphi }_{1}{\left(\frac{n-{\vartheta }_{1}}{{\vartheta }_{1}}\right)}^{k}+{\varphi }_{2}{\left(\frac{n-{\vartheta }_{2}}{{\vartheta }_{2}}\right)}^{k}\right\}}^\frac{1}{k}}\right)},{s}_{n\left(1- \frac{1}{1+{\left\{{\varphi }_{1}{\left(\frac{{\delta }_{1}}{n-{\delta }_{1}}\right)}^{k}+{\varphi }_{2}{\left(\frac{{\delta }_{2}}{n-{\delta }_{2}}\right)}^{k}\right\}}^\frac{1}{k}}\right)}\right)$$

Consequently, $$LIFDW{G}_{\varphi }\left({g}_{1},{g}_{2}\right)=\left({s}_{n\left(\frac{1}{1+{\left\{{\sum }_{i=1}^{2}{\varphi }_{i}{\left(\frac{n-{\vartheta }_{i}}{{\vartheta }_{i}}\right)}^{k}\right\}}^\frac{1}{k}}\right)},{s}_{n\left(1- \frac{1}{1+{\left\{{{\sum }_{i=1}^{2}{\varphi }_{i}\left(\frac{{\delta }_{i}}{n-{\delta }_{i}}\right)}^{k}\right\}}^\frac{1}{k}}\right)}\right)$$

Therefore, the result is valid when $q$ is equal to $2$. Let's assume that the result is valid for $q=p.$ Then, $LIFDW{G}_{\varphi }\left({g}_{1},{g}_{2},\dots ,{g}_{p}\right)={\otimes }_{i=1}^{p}\left({{g}_{i}}^{{\varphi }_{i}}\right)$. So,

$$LIFDW{G}_{\varphi }\left({g}_{1,}{g}_{2},...,{g}_{p}\right)=\left({s}_{n\left(\frac{1}{1+{\left\{{\sum }_{i=1}^{p}{\varphi }_{i}{\left(\frac{n-{\vartheta }_{i}}{{\vartheta }_{i}}\right)}^{\kappa }\right\}}^{\frac{1}{\kappa }}}\right)},{s}_{n\left(1- \frac{1}{1+{\left\{{\sum }_{i=1}^{p}{\varphi }_{i}{\left(\frac{{\delta }_{i}}{n-{\delta }_{i}}\right)}^{\kappa }\right\}}^{\frac{1}{\kappa }}}\right)}\right)$$

Now, consider the case for $q=p+1,$

$$LIFDW{G}_{\varphi }\left({g}_{1},{g}_{2},...,{g}_{p},{g}_{p+1}\right)={\otimes }_{i=1}^{p}\left({{g}_{i}}^{{\varphi }_{i}}\right)\otimes \left({{g}_{p+1}}^{{\varphi }_{p+1}}\right)$$

$$=\left({s}_{n\left(\frac{1}{1+{\left\{{\sum }_{i=1}^{p}{\varphi }_{i}{\left(\frac{n-{\vartheta }_{i}}{{\vartheta }_{i}}\right)}^{\kappa }\right\}}^{\frac{1}{\kappa }}}\right)},{s}_{n\left(1- \frac{1}{1+{\left\{{\sum }_{i=1}^{p}{\varphi }_{i}{\left(\frac{{\delta }_{i}}{n-{\delta }_{i}}\right)}^{\kappa }\right\}}^{\frac{1}{\kappa }}}\right)}\right)\otimes \left({s}_{n\left(\frac{1}{1+{\left\{{\varphi }_{p+1}{\left(\frac{n-{\vartheta }_{p+1}}{{\vartheta }_{p+1}}\right)}^{\kappa }\right\}}^{\frac{1}{\kappa }}}\right)},{s}_{n\left(1- \frac{1}{1+{\left\{{\varphi }_{p+1}{\left(\frac{{\delta }_{p+1}}{n-{\delta }_{p+1}}\right)}^{\kappa }\right\}}^{\frac{1}{\kappa }}}\right)}\right)$$

Consequently,

$$LIFDW{G}_{\varphi }\left({g}_{1,}{g}_{2},...,{g}_{p},{g}_{p+1}\right)=\left({s}_{n\left(\frac{1}{1+{\left\{{\sum }_{i=1}^{p+1}{\varphi }_{i}{\left(\frac{n-{\vartheta }_{i}}{{\vartheta }_{i}}\right)}^{\kappa }\right\}}^{\frac{1}{\kappa }}}\right)},{s}_{n\left(1- \frac{1}{1+{\left\{{\sum }_{i=1}^{p+1}{\varphi }_{i}{\left(\frac{{\delta }_{i}}{n-{\delta }_{i}}\right)}^{\kappa }\right\}}^{\frac{1}{\kappa }}}\right)}\right)$$

Thus, the result is applicable to $q=p+1.$ This completes the proof.

#### Example 3.

In instance 2, we manipulated a problem by the application of LIFDWA operator. Here let’s tackle the same issue by the utilization of LIFDWG operator.

$$LIFDW{G}_{\varphi }\left({g}_{1},{g}_{2},{g}_{3}\right)={\otimes }_{i=1}^{3}{{g}_{i}}^{{\varphi }_{i}}$$

$${\otimes }_{i=1}^{3}{{g}_{i}}^{{\varphi }_{i}}=\left({s}_{n\left(\frac{1}{1+{\left\{{\sum }_{i=1}^{3}{\varphi }_{i}{\left(\frac{n-{\vartheta }_{i}}{{\vartheta }_{i}}\right)}^{\kappa }\right\}}^{\frac{1}{\kappa }}}\right)},{s}_{n\left(1- \frac{1}{1+{\left\{{\sum }_{i=1}^{3}{\varphi }_{i}{\left(\frac{{\delta }_{i}}{n-{\delta }_{i}}\right)}^{\kappa }\right\}}^{\frac{1}{\kappa }}}\right)}\right)$$

Subsequently,$$LIFDW{G}_{\varphi }\left({g}_{1},{g}_{2},{g}_{3}\right)=\left({s}_{2.6977},{s}_{2.5462}\right).$$

#### Theorem 6.

*(Idempotency):* Consider $q$ number of LIFNs indicated as ${g}_{i}= \left({s}_{{\vartheta }_{i}},{s}_{{\delta }_{i}}\right),$ $i=\left(\text{1,2},...,q\right)$ defined on $\overline{S}=\left\{{s}_{\alpha }|\alpha \in \left[0,n\right]\right\}$ and $\varphi ={\left({\varphi }_{1},{\varphi }_{2},...,{\varphi }_{q}\right)}^{T}$ is the associated weight vector of ${g}_{i}$ with ${\varphi }_{i}>0$ and ${\sum }_{i=1}^{q}{\varphi }_{i}=1$. If all ${g}_{i}=\left({s}_{{\vartheta }_{i}},{s}_{{\delta }_{i}}\right)=\left({s}_{\vartheta },{s}_{\delta }\right)$ for any $i.$ Then;

$$LIFDW{G}_{\varphi }\left({g}_{1},{g}_{2},...,{g}_{q}\right)=\left({s}_{\vartheta },{s}_{\delta }\right).$$

#### Proof.

The proof is similar to that of theorem 2.

#### Theorem 7.

*(Boundedness):* Consider $q$ number of LIFNs indicated as ${g}_{i}= \left({s}_{{\vartheta }_{i}},{s}_{{\delta }_{i}}\right),$ $i=\left(\text{1,2},...,q\right)$ defined on $\overline{S}=\left\{{s}_{\alpha }|\alpha \in \left[0,n\right]\right\}$ and $\varphi ={\left({\varphi }_{1},{\varphi }_{2},...,{\varphi }_{q}\right)}^{T}$ is the associated weight vector of ${g}_{i}$ with ${\varphi }_{i}>0$ and ${\sum }_{i=1}^{q}{\varphi }_{i}=1$. Assume ${g}^{-}=\mathit{min}\left\{{g}_{1},{g}_{2},...,{g}_{q}\right\}$ and ${g}^{+}=\mathit{max}\left\{{g}_{1},{g}_{2},...,{g}_{q}\right\}$.Then ${g}^{-}\le LIFDW{G}_{\varphi }\left({g}_{1},{g}_{2},...,{g}_{q}\right)\le {g}^{+}.$

#### Proof.

The proof is similar to that of theorem 3.

#### Theorem 8.

*(Monotonicity):* Consider $q$ number of LIFNs indicated as ${g}_{i}= ({s}_{{\vartheta }_{i}},{s}_{{\delta }_{i}})$ and ${{g}_{i}}^{*}=\left({s}_{{\theta }_{i}},{s}_{{\sigma }_{i}}\right),$ $i=\left(\text{1,2},...,q\right)$ defined on $\overline{S}=\left\{{s}_{\alpha }|\alpha \in \left[0,n\right]\right\}$ and $\varphi ={\left({\varphi }_{1},{\varphi }_{2},...,{\varphi }_{q}\right)}^{T}$ is the associated weight vector of ${g}_{i}$ with ${\varphi }_{i}>0$ and ${\sum }_{i=1}^{q}{\varphi }_{i}=1$. If ${s}_{{\mu }_{i}}\le {s}_{{\theta }_{i}}$ and ${s}_{{\nu }_{i}}\ge {s}_{{\sigma }_{i}}.$ Then, ${LIFDWG}_{\varphi }\left({g}_{1},{g}_{2},\dots ,{g}_{q}\right)\le {LIFDWG}_{\varphi }\left({g}_{1}^{*},{g}_{2}^{*},\dots ,{g}_{q}^{*}\right)$.

#### Proof.

The proof is similar to that of theorem 4.

## Utilization of designed aggregation operators in MADM framework

In the subsequent section, we propose a decision making approach for MADM problems, where criteria weights are real numbers and criteria values are LIFNs. Consider $Q=\left\{{Q}_{1},{Q}_{2},...,{Q}_{p}\right\}$ be a collection of alternatives, and $R=\left\{{R}_{1},{R}_{2},...,{R}_{q}\right\}$ be the set of attributes. In addition, $\varphi ={\left({\varphi }_{1},{\varphi }_{2},...,{\varphi }_{q}\right)}^{T}$ represents the weight vector of attributes, where ${\varphi }_{i}\in [\text{0,1}]$ for all $i=\text{1,2},...,q$ such that ${\sum }_{i=1}^{q}{\varphi }_{i}=1.$ Consider a scenario in which the decision maker assesses the provided alternatives across different attributes and expresses their preference values using LIFNS denoted as ${g}_{ij}=\left({s}_{{\theta }_{ij}},{s}_{{\sigma }_{ij}}\right)$,where ${s}_{{\theta }_{ij}},{s}_{{\sigma }_{ij}}\in \overline{S}=\left\{{s}_{\alpha }|{s}_{0}\le {s}_{\alpha }\le {s}_{n},\alpha \in \left[0,n\right]\right\}.$ Afterwards, a LIF decision matrix $W={\left({g}_{ij}\right)}_{p\times q}$ is utilized to summarize the information supplied by the decision-maker.

The following are the major components of the suggested method for solving the MADM issues that is based on the LIFDWA and LIFDWG operators:

**Step 1.** Construct a LIF decision matrix $W={\left[{g}_{ij}\right]}_{p\times q}$ having entries as LIFNs corresponding to the given alternatives relative to the attributes.

**Step 2.** Calculate all the aggregated values ${g}_{i}=\left({s}_{{\vartheta }_{i}},{s}_{{\delta }_{i}}\right)$ of all the alternatives ${Q}_{i}$, where $i=\text{1,2},...,p$ in the framework of LIFDWA operator in the following way:

$${g}_{i}=LIFDWA\left({g}_{i1},{g}_{i2},...,{g}_{iq}\right)$$

$$=\left({s}_{n\left(1- \frac{1}{1+{\left\{{\sum }_{j=1}^{q}{\varphi }_{j}{\left(\frac{{\vartheta }_{ij}}{n-{\vartheta }_{ij}}\right)}^{\kappa }\right\}}^{\frac{1}{\kappa }}}\right)},{s}_{n\left(\frac{1}{1+{\left\{{\sum }_{j=1}^{q}{\varphi }_{j}{\left(\frac{n-{\delta }_{ij}}{{\delta }_{ij}}\right)}^{\kappa }\right\}}^{\frac{1}{\kappa }}}\right)}\right)$$

Likewise, the aggregated value of ${g}_{ij}$ within the context of LIFDWG operator is determined as follows:

$${g}_{i}=LIFDWG\left({g}_{i1},{g}_{i2},...,{g}_{iq}\right)=\left({s}_{n\left(\frac{1}{1+{\left\{{\sum }_{j=1}^{q}{\varphi }_{j}{\left(\frac{n-{\vartheta }_{ij}}{{\vartheta }_{ij}}\right)}^{\kappa }\right\}}^{\frac{1}{\kappa }}}\right)},{s}_{n\left(1- \frac{1}{1+{\left\{{\sum }_{j=1}^{q}{\varphi }_{j}{\left(\frac{{\delta }_{ij}}{n-{\delta }_{ij}}\right)}^{\kappa }\right\}}^{\frac{1}{\kappa }}}\right)}\right)$$

**Step 3.** Find the score values for all ${g}_{i}$, making use of definition 9.

**Step 4.** Create a scoring mechanism as specified in definition 9 to assess all the alternatives and ascertain the most optimal alternative.

A pseudocode representation of the techniques that have been devised is illustrated in Table 1.

**Table 1:**
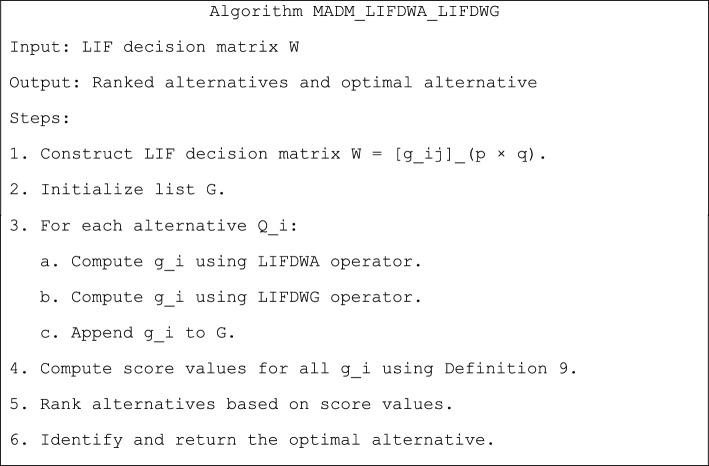
Pseudocode for the developed mechanisms.

### Case study-selecting an efficient bioremediation technique for cleaning contaminated soil

Clean soil is critical for a variety of reasons, including serving as the foundation for a healthy and sustainable environment^58^. Soil is an important substrate for plant growth because it provides needed nutrients and supports agriculture, which is critical for food supply^59^. In addition, healthy soil prevents precipitation and runoff from compromising clean water sources and ensures food security. Biological diversity, including terrestrial bacteria, plants, and animals, is influenced by soil health. Additionally, soil pollution can damage biological processes and natural ecosystems. This may raise serious health concerns. These impurities in the water might hinder crop growth and agricultural production. Robust plant development optimizes soil decontamination, which boosts crop production, agricultural sustainability, and food security over time. In the macro view, there is huge value in cleaning soils of chemical contaminants, both in terms of soil as an agent for conserving environmental quality, protecting human health, promoting sustainable agriculture, and preserving ecosystem well-being^60^. Soil pollution is still a global concern, as it can damage ecosystems and human health. As such, bioremediation is useful because it enlists living organisms (ranging from microbes to plants) to either absorb contaminants or break them down into less harmful forms. After enough time, the organisms can degrade or alter chemicals (say, pesticides or fertilizers) until they’re something a soil would naturally contain, which in turn reduces their harmful qualities to the point where they won’t hurt the land, crops, or otherwise spread through the environment. Studying the efficiency of different bioremediation techniques for cleaning contaminated soil is crucial for optimizing effectiveness, conducting comprehensive environmental impact assessments, analyzing cost-efficiency, and fostering technological advancements. This research ensures the development of targeted, sustainable, and impactful solutions to effectively manage and mitigate the adverse effects of soil pollution on ecosystems and human health.

### Exemplary illustration

This subsection presents a systematic approach for determining the optimal bioremediation method for remediating contaminated soil on a large scale within the constraints of LIF Dombi AOs.

Consider that petroleum byproducts and heavy metals from an abandoned industrial site in a bustling city have poisoned the earth. The local administration wants to turn this area into a vibrant community park. Thus, a team of experts was organized to clean up the contaminated area. The panel proposed four options to achieve this goal.

1. ${Q}_{1}:$ Phytoremediation, which uses plants to absorb toxins from soil,
2. ${Q}_{2}:$ Biostimulation is the process wherein soil nutrients assist in the degradation of pollutants by helping microorganisms.
3. ${Q}_{3}:$ Bioaugmentation accelerates pollutant breakdown in polluted soil by adding microorganisms
4. ${Q}_{4}:$ Mycoremediation, which uses fungi to breakdown and remove soil pollutants.

The group also took into account the following set of four criteria $\left\{{R}_{1},{R}_{2},{R}_{3},{R}_{4}\right\}$ that impact the effectiveness of these alternatives:

1. ${R}_{1}:$ Rate of contaminants removal;
2. ${R}_{2}:$ Soil quality improvement;
3. $${R}_{3}:$$ Environmental impact;
4. $${R}_{4}:$$ Time efficiency;

Furthermore, the set $S=\{{s}_{0}=$ extremely poor $,{s}_{1}=$ very poor $,{s}_{2}=$ poor $,{s}_{3}=$ slightly poor $,{s}_{4}=$ fair $,{s}_{5}=$ slightly good $,{s}_{6}=$ good $,{s}_{7}=$ very good $,{s}_{8}=$ extremely good $\}$ is chosen as a linguistic terms set to deal the problem in terms of LIF environment.

**Step 1**. The Table 2 explains the professional opinion of the decision-makers on each alternative ${Q}_{i},\left(i=1,...,4\right)$ corresponding to each attribute ${R}_{j},\left(j=1,...,4\right)$ in the form of LIFN.

**Table 2:** LIF decision matrix.

	$table$	${R}_{2}$	${R}_{3}$	${R}_{4}$
${Q}_{1}$	$\left({s}_{3},{s}_{4}\right)$	$\left({s}_{5},{s}_{3}\right)$	$\left({s}_{4},{s}_{3}\right)$	$\left({s}_{3},{s}_{4}\right)$
${Q}_{2}$	$\left({s}_{5},{s}_{2}\right)$	$\left({s}_{2},{s}_{1}\right)$	$\left({s}_{3},{s}_{4}\right)$	$\left({s}_{2},{s}_{5}\right)$
${Q}_{3}$	$\left({s}_{2},{s}_{1}\right)$	$\left({s}_{3},{s}_{2}\right)$	$\left({s}_{1},{s}_{2}\right)$	$\left({s}_{2},{s}_{3}\right)$
${Q}_{4}$	$\left({s}_{5},{s}_{2}\right)$	$\left({s}_{3},{s}_{3}\right)$	$\left({s}_{5},{s}_{2}\right)$	$\left({s}_{4},{s}_{1}\right)$

The decision-maker designates the weight vector as $\varphi ={\left(\text{0.20,0.40,0.30,0.10}\right)}^{T}$, with ${\sum }_{i=1}^{4}{\varphi }_{i}=1.$ In the subsequent discourse, we proceed to solve the decision matrix in order to ascertain the most efficient alternative through the utilization of the LIFDWA and LIFDWG operators.

### Part A

**Step 2.** The results of applying the LIFDWA operator to the values in Table 2 with a particular value of $\kappa =3$ are as shown in Table 3.

**Table 3:** Combined scores of alternatives using the LIFDWA operator.

Alternatives	${{\varvec{g}}}_{{\varvec{i}}}$
${Q}_{1}$	$\left({s}_{4.5276},{s}_{3.1694}\right)$
${Q}_{2}$	$\left({s}_{4.0061},{s}_{1.2844}\right)$
${Q}_{3}$	$\left({s}_{2.5267},{s}_{1.4686}\right)$
${Q}_{4}$	$\left({s}_{4.6065},{s}_{1.7111}\right)$

**Step 3.** By the application of definition 9, compute the score values for all LIFNs obtained in step 2. $S\left({Q}_{1}\right)={s}_{1.4336},S\left({Q}_{2}\right)={s}_{1.7851},S\left({Q}_{3}\right)={s}_{2.0010},S\left({Q}_{4}\right)={s}_{1.6492}.$

**Step 4.** Since $\text{S}\left({\text{Q}}_{3}\right)>\text{S}\left({\text{Q}}_{2}\right)>\text{S}\left({\text{Q}}_{4}\right)>\text{S}\left({\text{Q}}_{1}\right),$ therefore, the ranking order of alternatives is ${Q}_{3}>{Q}_{2}>{Q}_{4}>{Q}_{1}.$

Consequently, in view of the ranking system, we conclude that Bioaugmentation is the most efficient Bioremediation technique for cleaning contaminated soil.

### Part B

**Step 2.** The results of applying the LIFDWG operator to the values in Table 2 with a particular value of κ = 3 are as shown in Table 4.

**Table 4:** Combined scores of alternatives using the LIFDWG operator.

Alternatives	${{\varvec{g}}}_{{\varvec{i}}}$
${Q}_{1}$	$\left({s}_{3.6185},{s}_{3.4725}\right)$
${Q}_{2}$	$\left({s}_{2.3105},{s}_{3.8272}\right)$
${Q}_{3}$	$\left({s}_{1.3716},{s}_{2.1336}\right)$
${Q}_{4}$	$\left({s}_{3.5206},{s}_{2.5655}\right)$

**Step 3.** By the application of definition 9, compute the score values for all LIFNs obtained in step 2.$S\left({Q}_{1}\right)={s}_{1.4499},S\left({Q}_{2}\right)={s}_{1.5041},S\left({Q}_{3}\right)={s}_{2.1468},S\left({Q}_{4}\right)={s}_{1.6221}.$

**Step 4.** Since $S\left({Q}_{3}\right)>S\left({Q}_{4}\right)>S\left({Q}_{2}\right)>S\left({Q}_{1}\right),$ therefore, the ranking order of alternatives is ${Q}_{3}>{Q}_{4}>{Q}_{2}>{Q}_{1}.$

Consequently, in view of the ranking system, we conclude that Bioaugmentation is the most efficient Bioremediation technique for cleaning contaminated soil.

### Comparative analysis

The purpose of this subsection is to provide a comprehensive assessment that demonstrates the significance and dependability of the proposed model. This comparative analysis employs established methodologies to assess multiple alternatives. We examine the LIFDWA and LIFDWG operators in this discourse with the more widely recognized operators that were developed in ^11–13,43^ (See Tables 5, 6).

**Table 5:** Aggregated values obtained from different existing operators.

Alternative	LIFWA^43^	LIFWG^43^
${{\varvec{Q}}}_{1}$	$\left({s}_{4.1880},{s}_{3.2704}\right)$	$\left({s}_{4.0118},{s}_{3.3238}\right)$
${{\varvec{Q}}}_{2}$	$\left({s}_{3.0547},{s}_{2.0451}\right)$	$\left({s}_{2.7130},{s}_{2.7277}\right)$
${{\varvec{Q}}}_{3}$	$\left({s}_{2.1580},{s}_{1.8131}\right)$	$\left({s}_{1.9105},{s}_{1.9239}\right)$
${{\varvec{Q}}}_{4}$	$\left({s}_{4.2125},{s}_{2.1946}\right)$	$\left({s}_{3.9860},{s}_{2.3353}\right)$

**Table 6:** Ranking of alternatives based on score values.

Operators	${{\varvec{Q}}}_{1}$	${{\varvec{Q}}}_{2}$	${{\varvec{Q}}}_{3}$	${{\varvec{Q}}}_{4}$	Ranking
LIFWA ^43^	${s}_{1.4411}$	${s}_{1.7824}$	${s}_{2.0070}$	${s}_{1.6125}$	${Q}_{3}>{Q}_{2}>{Q}_{4}>{Q}_{1}$
LIFWG ^43^	${s}_{1.4452}$	${s}_{1.6943}$	${s}_{2.0423}$	${s}_{1.6122}$	${Q}_{3}>{Q}_{2}>{Q}_{4}>{Q}_{1}$
LIFDWA	${s}_{1.4336}$	${s}_{1.7851}$	${s}_{2.0010}$	${s}_{1.6492}$	${Q}_{3}>{Q}_{2}>{Q}_{4}>{Q}_{1}$
LIFDWG	${s}_{1.4499}$	${s}_{1.5041}$	${s}_{2.1468}$	${s}_{1.6221}$	${Q}_{3}>{Q}_{4}>{Q}_{2}>{Q}_{1}$

- It is crucial to note that the decision-making situation described above cannot be effectively addressed by the techniques proposed by Xu^11^, Zhao^12^, and Xu^13^. While these methodologies establish membership and non-membership through numerical degrees, which produce precise results but may pose interpretability challenges, the suggested approaches utilize language concepts to represent these degrees, thereby enhancing interpretability. In order to incorporate linguistic modifiers (such as "very" and "slightly") and linguistic terms into AOs for LIFS, non-numerical manipulation techniques are necessary. These approaches prioritize interpretability over exactitude in numbers, which aligns more closely with the cognitive processes of humans in the LIF environment. Furthermore, it should be noted that the approaches outlined in^11–13^ fail to address challenges that place significant importance on qualitative and human-centered factors. However, the recently suggested methods are advantageous as they provide a means to reconcile the disparities between intuitive fuzzy numbers and language-comprehensible expressions.
- From the above table, it is quite evident that our articulated operators, namely LIFDWA and LIFDWG operators designed for MADM problems, offer heightened accuracy and precision in comparison to LIFWA and LIFWG operators^43^. The LIFWA and LIFWG operators defined in^43^ do not have the flexibility to deal with MADM problems using parametric values. Due to this drawback, these mathematical models lack flexibility and adaptability in different complex scenarios. Within our devised operators, the introduction of an operational parameter imparts the capacity to adjust preference values associated with specific decision requirements. This affords decision-makers the flexibility to select an optimal parameter value aligned with their individual risk tolerance and contextual demands.

Diverging from established methodologies, our approaches excel at managing scenarios characterized by closely intertwined arguments, rendering our proposed technique universally applicable. The conceptual framework introduced in this article is uniquely tailored for the LIF environment, a distinction from prevailing methods that predominantly address fuzzy information and linguistic variables but are not as flexible as in the domain of Dombi AOs defined in this article under the LIF environment.

Hence, we conclude that this article presents innovative approaches for resolving decision-making problems, showcasing the flexibility of the proposed operators through parameter modulation. This characteristic not only enhances the adaptability of the operators but also provides decision-makers with a nuanced spectrum of choices for evaluating decisions, thus amplifying the versatility and applicability of the proposed operators in the context of practical decision-making scenarios.

## Conclusions

The realm of MADM has witnessed the continual evolution of various methodologies to address complex real-world challenges. Recent scholarly contributions have yielded an array of sophisticated techniques. This research work has been dedicated to the introduction of two novel LIF Dombi AOs, namely, LIFDWA operator and LIFDWG operator. Fundamental structural properties of these operators have succinctly been investigated. In addition, for the resolution of MADM issues within the LIF framework, a new score function has been developed. Moreover, a mathematical approach has been designed to address MADM challenges using the proposed techniques. Furthermore, these methodologies have effectively been implemented to discern the optimal bioremediation technique for the purification of contaminated soil. This application serves as empirical evidence affirming the authenticity and heightened efficacy of our proposed methodologies when compared with existing approaches.

### Limitations of the proposed methodology

1. Although the strategies outlined in this research work present numerous benefits, they are not devoid of constraints. It is important to highlight that they are limited in circumstances where the combined degrees of linguistic membership and non-membership exceed $[0,n].$
2. The presented approach may not be suitable for situations involving data capture at various time intervals due to the absence of an intrinsic dynamic adjustment mechanism for MADM challenges.

### Future research directions

1. In our future studies, we will also expand the scope of the recently defined techniques to more comprehensive academic fields like linguistic Pythagorean fuzzy, linguistic interval-valued fuzzy, and linguistic IF dynamic environments to address the shortcomings of the present research.
2. We will also explore the adaptability and relevance of our approach across various decision-making fields, such as healthcare, environmental impact evaluation, and natural disaster response strategies.

## Data availability 

All data generated or analyzed during this study are included in this article.
